# Effects of worksite health promotion interventions on employee diets: a systematic review

**DOI:** 10.1186/1471-2458-10-62

**Published:** 2010-02-10

**Authors:** Cliona Ni Mhurchu, Louise M Aston, Susan A Jebb

**Affiliations:** 1 MRC Human Nutrition Research, Cambridge, UK

## Abstract

**Background:**

Public health strategies place increasing emphasis on opportunities to promote healthy behaviours within the workplace setting. Previous research has suggested worksite health promotion programmes have positive effects on physical activity and weight loss, yet little is known regarding their effects on dietary behaviour. The aim of this review was to assess the effects of worksite interventions on employee diets.

**Methods:**

Electronic databases (MEDLINE, The Cochrane Library, PsycINFO, EMBASE, LexisNexis) were searched for relevant articles published between 1995 and April 2009. Studies were eligible for inclusion if they were peer-reviewed English language publications describing a worksite-based health promotion intervention with minimum study duration of eight weeks. All study designs were eligible. Studies had to report one or more diet-related outcome (energy, fat, fruit, or vegetable intakes). Methodological quality was assessed using a checklist that included randomisation methods, use of a control group, and study attrition rates.

**Results:**

Sixteen studies were included in the review. Eight programmes focussed on employee education, and the remainder targeted change to the worksite environment, either alone or in combination with education. Study methodological quality was moderate. In general, worksite interventions led to positive changes in fruit, vegetable and total fat intake. However, reliance on self-reported methods of dietary assessment means there is a significant risk of bias. No study measured more robust outcomes such as absenteeism, productivity, or healthcare utilisation.

**Conclusions:**

The findings of this review suggest that worksite health promotion programmes are associated with moderate improvement in dietary intake. The quality of studies to date has been frequently sub-optimal and further, well designed studies are needed in order to reliably determine effectiveness and cost-effectiveness. Future programmes to improve employee dietary habits should move beyond individual education and aim to intervene at multiple levels of the worksite environment.

## Background

Poor nutrition is an important contributor to several serious health conditions, such as type 2 diabetes, cardiovascular disease, and many common cancers [1]. Estimations of the global burden of disease attributable to nutrition-related risk factors (excess body weight, low fruit and vegetable intakes, high blood pressure and high blood cholesterol levels) demonstrates that they are leading causes of loss of healthy life, causing approximately 17 million deaths and over 160 million lost years of healthy life in 2000 [1]. The economic burden is also significant with food-related ill-health estimated to cost the National Health Service (NHS) about £6 billion annually [2]. The consequences of poor diet and excess body weight impact directly on employers, with obesity being one of the most common and costly health problems encountered at work, and many others (back pain, stress, coronary heart disease and diabetes) are causally linked to poor diet and obesity [3]. Obese people also suffer more sickness and absences from work [4], with around 16 million lost working days attributable to obesity-related illness in the UK in 2002 [5].

Achieving a healthy workforce should therefore not only result in improved health for individuals, but also bring benefits to employers and society. In addition to reducing absenteeism, worksite initiatives to promote health and well-being lead to economic benefits for businesses [6]. Since individuals spend up to 60% of their waking hours in their place of work [3], worksite interventions have significant potential to improve dietary habits and promote weight loss. In addition, effective interventions may lead to secondary improvements in lifestyles of employees and their families outside of the worksite.

A healthy weight workforce may also help create a positive corporate image. This is particularly relevant to the NHS, where many employees are involved directly in advising the general public about health. The Cabinet Office Strategy Unit estimates that, if representative of the UK working population as a whole, the NHS may have almost one million overweight and obese staff [7]. Research has revealed that overweight people question the validity of advice given by overweight health professionals [8]. Therefore, achieving better health for health professionals may have indirect benefits for patients.

Both diet and physical activity are important in achieving and maintaining a healthy body weight. Improvements to dietary intakes also confer important benefits to health beyond maintenance of a healthy body weight [9,10]. A substantial body of research has been undertaken in relation to promoting weight loss [11,12] and increasing physical activity opportunities [13-15] in the worksite, but much less is known regarding the effects of such interventions on dietary habits. The aim of this systematic review was therefore to assess the effects of worksite interventions on dietary outcomes.

## Methods

### Search strategy

This review was undertaken as part of a larger review of the effects of worksite interventions to improve diet and promote weight loss, commissioned by locality obesity groups. Since recent reviews have described the impact of worksite interventions on weight loss [11,12], we chose to focus on diet for this paper since less is known about the impact of such programmes on this outcome.

A search was undertaken for all worksite health promotion studies with dietary outcomes published after 1994. The following electronic databases were searched up to April 2009 for relevant peer-reviewed articles: MEDLINE, The Cochrane Library, PsycINFO, EMBASE, and LexisNexis. The following MESH search terms were used for MEDLINE and adapted slightly for use with other databases: worksite, workplace, occupational health, body weight, body weight changes, weight loss, obesity, overweight, body mass index (BMI), diet, diet therapy, nutrition therapy, nutrition policy, food services. The reference lists of relevant studies and review articles were also hand searched. The search dates, databases, and search terms were chosen to maintain consistency with an earlier review of worksite weight loss programmes [12].

### Study selection and data extraction

Studies were eligible for inclusion in the review if they were published, peer-reviewed, English language articles describing a worksite-based weight loss and/or healthy eating intervention with a minimum study duration of eight weeks. All study designs were eligible. To be eligible for inclusion, articles had to report one or more dietary outcomes (e.g. energy, fat, fruit or vegetable intakes) assessed using an accepted, validated method of dietary assessment at eight weeks or later following baseline. The review was restricted to English language articles. One author (LMA) reviewed the titles, abstracts and keywords of every record retrieved and the full article was retrieved for further assessment if available information suggested that the study was eligible for inclusion. A standardised data extraction form was then completed for all eligible studies. Data were recorded on country of origin, type of worksite, participant characteristics, study design, intervention characteristics, study outcome measures, and reported results. Study quality was assessed using a checklist adapted from a previous review [12] and included assessment of use of a control group, randomisation, and study attrition rates. For controlled trials, assessment was made of concealment of randomised allocation, blinding, use of intention-to-treat analyses, and similarity of baseline characteristics of participants.

## Results

**Figure 1:**
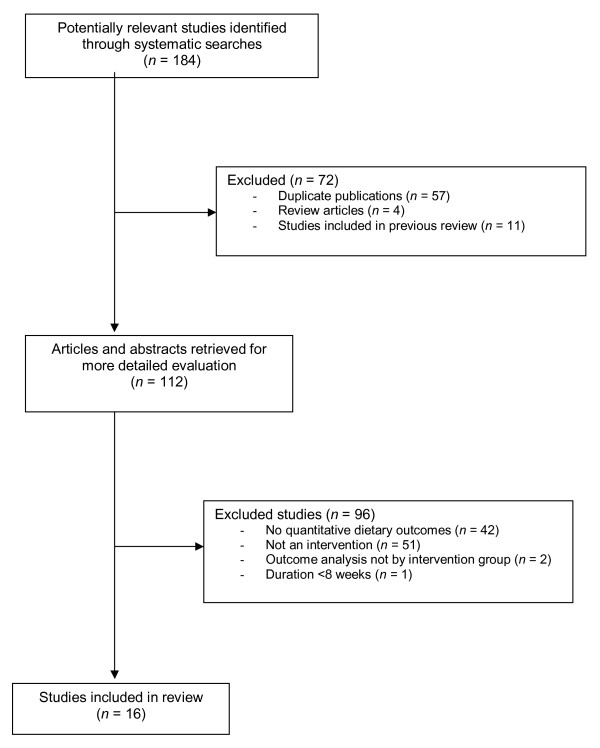
**Results of systematic search and reasons for study exclusion**.

One hundred and eighty four potentially relevant studies were identified, of which 112 were retrieved for detailed evaluation. Following exclusion of ineligible studies, 16 were included in the review (Figure 1).

Additional File 1 summarises the characteristics of the 16 included studies. Study participants varied considerably by gender (0-100% male) and health status. Mean age of participants in individual studies ranged from 38 to 49 years, and a variety of worksite settings were represented. One international study involved worksites in 17 countries [16]. More than half were undertaken in North America (n = 9) [17-25], and the remainder were from Europe (n = 6) [26-31]. Study sizes ranged from 84 to 5,156 employees, and from one to 84 worksites.

### Interventions

Programme interventions were described in variable detail. Eight studies (50%) implemented programmes focussing on employee education [16-20,26,27,30]; two (13%) targeted changes to worksite policy and/or environment; and six (37%) employed a combination of education and environmental changes [21-25,32,33]. Strategies to deliver education to employees included group and/or individual counselling, shopping tours, individual diet plans, computer-tailored dietary feedback, weekly health promotion email messages, and worker participation in programme planning. Environmental interventions utilised comprised changes to worksite nutrition policies and practices such as nutrition labelling, vending policies, canteen food supply/availability, and menu reformulation. Duration of follow-up ranged from 12 weeks to 2.5 years.

### Methodological quality

The methodological quality of the studies is summarised in Additional File 2. Ten studies (63%) were randomised controlled trials (RCTs) [17,20-27,33], one (6%) was a quasi-experimental study with a non-randomised comparison group [29], and five (31%) were uncontrolled intervention studies (pre-test post-test design) [16,18,19,28,30]. Nine RCTs (90%) undertook randomisation by worksite, while one randomised individual employees. Only one trial that randomised by worksite also conducted their analysis by worksite [20]; the remainder conducted analysis by individual employees. Randomisation resulted in similar baseline characteristics between intervention groups for five RCTs, but intervention groups were clearly different at baseline for the remainder. Most RCTs failed to report whether treatment allocation had been adequately concealed (9/10), if outcome assessors were blinded to treatment allocation (10/10), and if intention-to-treat analyses were conducted (7/10). The quality of quasi-experimental studies and uncontrolled intervention studies was clearly inferior to that of RCTs. Their lack of randomised, comparable control groups meant it was not possible to attribute any effects reported directly to the intervention, rather than merely trial participation and/or secular trends. Retention rates overall (the number of randomised participants or worksites who completed individual study follow-up) ranged from 21% to 100%.

### Dietary results

Study outcomes are reported in Additional File 3. There was substantial variability in aspects of diet examined and methods of dietary assessment used. The most common methods of dietary assessment were food frequency questionnaires and eating habits/dietary practices questionnaires. Lesser-used methods included 24-hour dietary recall, seven-day diet recall, food diary, worksite canteen sales data, and weighed measurement of worksite lunches.

Twelve studies measured fruit/vegetable intakes and nine measured total fat intakes. Four studies reported effects separately for fruit and vegetable intakes while the remainder combined fruit and vegetable intakes into a single outcome. Although all studies used daily servings as the unit measure for fruit and vegetables, two reported proportional change from baseline, one reported proportional change in meeting the target of 5 servings per day, while others reported absolute change in daily servings (5), daily intake in grams (2), or daily frequency of consumption (2). In two RCTs that measured proportional change in combined fruit and vegetable intakes, average daily increases ranged from +3% to +16% in intervention groups compared with -2% to +4% in control groups.

Most studies that measured total fat intake reported effects on percent energy from total fat but a small number reported results in grams per day, daily fat points, frequency of consumption of high fat foods, or dietary fat scores. In almost all studies, reported improvements in diet quality were greater in intervention groups compared with controls. In five RCTs that measured total fat as a percent of energy by intervention group, average daily reductions ranged from -2.2% to -9.1% in intervention groups compared with to +1.3% to -1.8% in control groups.

### Anthropometric results

Only three of the 16 studies also reported effects on body weight. In two, weight loss results were broadly consistent with reported dietary changes [17,19]. In an RCT, the intervention group reported greater reductions than the control group in dietary energy (-580 kcal/day versus -119 kcal/day) and fat (-6.7% versus +1.3%) intake and also achieved greater weight loss (-4.4 kg versus -1.0 kg)[17]. In the uncontrolled intervention study, participants decreased their fat score by 2.3 and also lost approximately 3 lbs in body weight [19]. However, in another RCT the intervention group increased their BMI more than the control group (+0.26 kg/m^2^), despite reporting greater reductions in energy (-142 kcal/day) and total fat (-1.6% energy) intakes [26]. In general, the effects of worksite interventions on diet were positive but the self-reported nature of dietary assessment means there is a substantial risk of bias.

### Economic results

No study included in the review measured the effect of worksite interventions on employee absenteeism, productivity and/or healthcare costs.

### Environmental interventions

A relatively small number of studies evaluated the effectiveness of worksite environmental interventions alone [28,29] or in combination with health education [21-25,33]. Findings of these eight studies were generally positive for dietary outcomes but effect sizes were small. Direct comparison with the eight studies that evaluated employee education interventions is difficult due to variability in study design and outcome measures, but typically individual-level interventions appeared to deliver slightly greater effects than environmental interventions.

## Discussion

The findings of this systematic review suggest that worksite interventions are effective in improving some measures of dietary behaviour. Effect sizes are variable but are generally small, although decreases of up to 9% in total dietary fat and increases up 16% in daily fruit and vegetable intakes have been reported. However, worksite intervention research has typically been methodologically weak and many studies have not included appropriately matched control groups, meaning reported effects may be due to trial participation rather than the actual worksite intervention programme. The use of self-reported dietary outcomes in most studies is a particular cause for concern because reporting bias due to dietary education makes it probable that effects on diet are over-estimated.

Our findings are fairly consistent with two recent systematic reviews of worksite weight loss interventions on body weight [11,12]. Benedict and Arterburn reviewed 11 intervention studies published between 1995 and 2006 and reported that intervention groups lost -0.2 to -6.4 kg more body weight than controls over follow-up periods ranging from two to 18 months [12]. Anderson et al reviewed 47 intervention studies published between 1966 and 2005, and a meta-analysis of a sub-set of nine RCTs produced a pooled effect estimate of -2.8 pounds of weight loss (95% confidence interval -4.6, -1.0) over 6-12 months of follow-up [11]. Thus, it appears that worksite health promotion interventions also have positive effects on employee body weight but effect sizes are small.

A 2005 review of 13 worksite programmes with environmental changes concluded there was strong evidence for an effect on dietary intake but inconclusive evidence for an effect on physical activity and no evidence for an effect on health risk factors [34]. The purported strongest evidence was for diet despite the fact that all dietary outcomes were self-reported rather than objectively measured. Similarly dietary outcomes in our review were predominantly self-reported, except in three of the 16 studies where sales data were used to supplement self-reported dietary changes [28,32,33]. There is an urgent need for future worksite dietary intervention studies to include objective measures of dietary behaviour and environments. Examples of such objective measures include body weight, biological risk factor levels such as blood cholesterol, canteen and/or vending machine sales data, and nutritional analysis of foods available at worksites. Similar recommendations have been made with respect to evaluation of worksite physical activity interventions [14]. Future studies should also consider assessing dietary intake outside the workplace because of the potential for compensatory behaviours elsewhere.

Previous reviews have highlighted the lack of long-term data on the effect of worksite health promotion programmes on health and economic outcomes [12,13,35]. Although some studies in our review had a relatively long duration of follow-up (up to 2.5 years), none reported effects on economic outcomes. Assessment of health and economic outcomes in worksite health promotion interventions should be a priority for future research, particularly given the advent of statistical methods that facilitate estimation of effects of changes in nutrition-related risk factors on burden of disease [10,36], and cost-effectiveness of interventions [37]. A recent review of the effects of worksite interventions on body weight reported that such programmes appear cost-effective and have the potential to boost profits of employers by increasing employee productivity and reducing medical care and costs [11]. However, robust evidence is still lacking.

The conduct of worksite-based research studies is clearly challenging. It frequently proves difficult to combine the need for academic rigour with the practicalities of delivering a community-based intervention that must meet employer and employee needs, often within short timeframes and constrained budgets. Nevertheless it is important that rigorous, independent, long-term evaluation of worksite health promotion initiatives occurs if we are to reach definitive conclusions about how effects on employee behaviour change translate into hard outcomes such as changes in body weight, health risks, healthcare utilisation, absenteeism, and productivity.

This review provides a comprehensive assessment of the impact of worksite interventions published over the past 15 years on dietary outcomes. It complements previous reviews that examined the impact of worksite interventions on physical activity [13-15] and weight loss [9,10] outcomes. Strengths include the systematic approach to searching the literature and inclusion of a broad range of study designs. Inclusion of study designs other than RCTs is important when evaluating complex interventions such as worksite programmes because application of an RCT design may be difficult and/or ethically inappropriate in practice. Limitations of the review include restriction of the search to studies published in English and use of a limited number of electronic databases. These search restrictions may account for the predominance of North American studies retrieved. However this may also be due to the fact that employer health insurance contributions are common in the United States, providing a greater incentive for US employers to implement and evaluate the effectiveness of worksite health promotion programmes. Publication bias may also mean some relevant worksite health promotion programmes were not included. This is a particular possibility with community health promotion initiatives where many non-academic schemes are not evaluated and/or published.

Public health strategies are placing increasing emphasis on the key role worksites can play in preventing illness and promoting health and well-being [6]. However, this review highlights a critical lack of evidence regarding the most acceptable and cost-effective worksite health programmes. Strategies employed to promote healthy eating to date have largely focussed on individual responsibility (education and behaviour change). Some programmes have implemented changes to worksite environments in order to make healthy choices easier but these have largely focussed on changing the physical environment, i.e. food availability, and have mostly failed to tackle the economic, political, and socio-cultural aspects of the worksite. Greater use of frameworks for interventions that acknowledge the complexity of the environment and the need to intervene at many levels may help to achieve more meaningful changes [38]. In particular, workplace canteens which frequently include a degree of food subsidisation provide an ideal environment in which to test the potential of economic incentives to change food purchasing behaviour [39]. Evidence suggests that economic incentives impact positively on dietary behaviour [40]; and favorable effects have been seen for weight loss [41,42], purchase of low-fat snacks [43], and self-reported fruit and vegetable consumption [44]. Changes to political (the rules) and socio-cultural (social norms) aspects of the worksite also merit more consideration in future interventions.

Before worksite programmes can be implemented with confidence and rolled out on a large scale, more social and behavioural research is needed to help identify determinants of eating habits and predictors of uptake of worksite health promotion programmes. Some worksite programmes have been based on solid groundwork exploring factors influencing potential programme adoption and implementation [31], but there remains a clear need to integrate qualitative and quantitative research methods in order to better evaluate reasons for success or failure of such complex interventions [45].

There is also a need to radically improve the quality and reporting of worksite intervention studies. Many published studies suffer from design flaws including the absence of a comparison group, reporting of multiple outcomes in the absence of a pre-specified study hypothesis and primary outcome, lack of objective outcome measures, and inappropriate statistical analyses.

## Conclusions

The findings of this review suggest that worksite interventions have a positive, but small, effect on dietary behaviour. The quality of worksite studies is however often sub-optimal and further, well designed studies are needed in order to reliably determine their effectiveness and cost-effectiveness. Such studies should include well-matched comparison groups, objective measures of environmental and individual dietary change, and sufficiently long periods of follow-up to determine long-term effects of programmes on employee health, absenteeism and productivity. Future programmes to improve employee dietary habits should aim to intervene at multiple levels of the worksite environment, particularly with respect to economic levers to influence food choice; and integrate qualitative methods with traditional study designs in order to provide more insight into reasons for programme success or failure.

## Competing interests

The authors declare that they have no competing interests.

## Authors' contributions

*Review conception and design*: LMA, CNM, SAJ; *Literature searching and data extraction*: LMA; *Analysis and interpretation of data*: CNM; *Drafting of the manuscript*: CNM; *Critical revision of the manuscript for important intellectual content*: CNM, LMA, SAJ. All authors read and approved the final manuscript.

## Pre-publication history

The pre-publication history for this paper can be accessed here:

http://www.biomedcentral.com/1471-2458/10/62/prepub

## Supplementary Material

Additional file 1 Characteristics of Included Studies Click here for file

Additional file 2 Quality of Included Studies Click here for file

Additional file 3 Dietary and Anthropometric Outcomes of Included Studies Click here for file
