# Supplementary material for: Effects of worksite health promotion interventions on employee diets: a systematic review
Source: BMC Public Health. 2010 Feb 10;10:62. doi: 10.1186/1471-2458-10-62 (PMC2829502; doi:10.1186/1471-2458-10-62)
Supplement: Additional file 1 — Characteristics of Included Studies [file 1471-2458-10-62-S1.PDF]

Table 1 - Characteristics of Included Studies

Randomised, controlled trials (n=10)

	Author, year, country	Participants & setting	Trial design	Follow-up	Intervention(s) and control	Anthropometric & dietary outcomes
1	Aldana, 2005<br>USA	145 employees (and spouses) of a medical care provider, 14% male, mean age 46 yr	Individual RCT	26 weeks	4-week intervention: weekly health education lectures, + access to shopping tours, cooking demos, and diet plans<br><br>Control: delayed intervention	Body weight, BMI, % body fat, dietary intake (Block FFQ)
2	Braekman, 1999<br>Belgium	638 male employees from 4 worksites, 100% male, mean age 44 yr	Cluster RCT<br><br>*UOR:*<br>Worksite<br><br>*UOA:*<br>Individual employees	13 weeks	3-month screening and education programme – group and individual counselling and education resources<br><br>Control: screening results summary without further information/advice to change dietary habits	BMI, WHR, dietary intake (24h recall), nutrition knowledge
3	Campbell, 2002<br>USA	859 rural female blue-collar employees at 9 worksites (*analysis restricted to 538 who completed 3 surveys*), 0% male, 53% aged ≤40 yr	Cluster RCT<br><br>*UOR:*<br>Worksite<br><br>*UOA:*<br>Worksite	78 weeks	18-month intervention: individualised computer tailored feedback and strategies for change + training of workers to diffuse information and provide support<br><br>Control: delayed intervention. Received one individually tailored feedback magazine after 6 months	BMI, total fat and F&V intakes (FFQ)
4	De Bourdeaudhuij, 2007<br>Belgium	539 employees of 6 companies (*analysis restricted to 337 who completed 2 surveys*), 32% male, mean age 39 yr	Cluster RCT<br><br>*UOR:*<br>Worksite<br><br>*UOA:*<br>Individual employees	26 weeks	One-off interactive computer-tailored fat reduction feedback intervention (completed within 14 days of sign-up)<br><br>Control 1: printed generic (non-tailored) fat reduction advice<br>Control 2: no intervention	Total fat intake (FFQ)

5	Emmons, 1999<br>USA	2055 employees at 22 worksites (*analysis restricted to 397 who completed min 2 surveys*), 56% male, mean age 42 yr	Cluster RCT<br><br>*UOR:*<br>Worksite<br><br>*UOA:*<br>Individual employees	130 weeks	2.5 yr multiple risk factor education programs + changes to nutrition policies and practices (labelling, vending, food availability)<br><br>Control: standard care (self-help programs)	Total fat, fibre, F&V intakes (FFQ)
6	Sorensen 1998<br>USA	2386 employees at 24 blue collar worksites, 76% male	Cluster RCT<br><br>*UOR:*<br>Worksite<br><br>*UOA:*<br>Individual employees	104 weeks	2-yr intervention: worker participation in program planning + individual behaviour change programmes + worksite environment changes (availability of healthy food)<br><br>Control: not described	Fibre, total fat, F&V intakes (FFQ)
7	Sorensen, 1999<br>USA	1359 employees at 22 community health centres (*1306 at follow-up – independent samples*), 16% male	Cluster RCT<br><br>*UOR:*<br>Worksite<br><br>*UOA:*<br>Individual employees	104 weeks	2-yr intervention<br><br>Intervention 1: worksite only (worker participation in program planning + individual behaviour change programmes<br>Intervention 2: worksite plus family intervention<br><br>Control: minimal intervention	F&V intake (FFQ)
8	Sorensen, 2003<br>USA	5156 employees at 15 manufacturing worksites	Cluster RCT<br><br>*UOR:*<br>Worksite<br><br>*UOA:*<br>Individual employees	104 weeks	2-yr worksite intervention (worker participation in program planning + individual behaviour change programmes + worksite environment changes) plus occupational health & safety intervention<br><br>Control: worksite only intervention	Dietary intake (F&V screening questionnaire)
9	Sorensen 2007<br>USA	974 employees at 24 small manufacturing worksites, 66% male, mean age 44 yr	Cluster RCT<br><br>*UOR:*	78 weeks	18-month intervention: individual behaviour change programme + worksite environment changes	F&V intake (F&V screening questionnaire)

			Worksite<br><br>*UOA:* Individual employees		Control: minimal intervention	
10	Steenhuis, 2004 Netherlands	1013 white collar employees at 17 worksites, 62% male, mean age 38 yr	Cluster RCT<br><br>*UOR:* Worksite<br><br>*UOA:* Individual employees	26 weeks	6-month intervention<br><br>Intervention 1: education only<br>Intervention 2: food supply + education<br>Intervention 3: labelling + education<br><br>Control: no intervention	Total fat, F&V intakes (FFQ), last meal at cafeteria, sales data for targeted product categories

Quasi-experimental studies (n=1)

	Author, year, country	Participants & setting	Trial design	Duration	Intervention(s)	Outcomes measured
1	Holdsworth, 2004 UK	577 employees at 6 worksites	QES (4 intervention, 2 control sites)	52 weeks	6-month intervention: change menus to >1/3 healthy choices + decrease fat, sugar, salt, increase fibre<br><br>Control: Workplaces who applied for but did not receive the Heartbeat award	Dietary habits (FFQ)

Uncontrolled intervention studies (pre-test post-test design) (n=5)

	Author, year, country	Participants & setting	Trial design	Duration	Intervention(s)	Outcomes measured
1	Block, 2004 USA	84 corporate worksite employees (*37% response rate*), 27% male, age range 21-63 yr	PTPT	12 weeks	12 weekly email messages tailored to usual diet: focus on increasing F&V and decreasing fat intakes	F&V intakes and sources of fat (FFQ)
2	Calderon, 2008 USA	366 space centre employees, 75% male, mean age 49 yr	PTPT	21 weeks	CVD risk reduction program – health education phone counselling (one phone call delivered one week post-screening)	Body weight, BMI, dietary fat intake (FFQ)
3	Holdsworth, 1999	12 worksite canteens with	PTPT	26-156	Heartbeat award scheme - change menus	Types of food sold in

	UK	Heartbeat awards at least 6 months previously		weeks	to > 1/3 healthy choices + decrease fat, sugar, salt, increase fibre + labelling of healthy options	canteens, sales of healthier food items
4	Lassen, 2003 Denmark	909 employees at 5 worksites, 25-71% male	PTPT	52 weeks	8-hr canteen staff training, employee dietary goal setting over 2 months	F&V intakes at lunch (net weight)
5	Pratt, 2006 17 countries	2498 General Electric employees, 77% male, mean age 42 yr	PTPT	52 weeks	Monthly emails, web-based nutrition coaches, newsletters	Body weight, BMI (self-reported), F&V intakes (questionnaire)

RCT: Randomised controlled trial
UOR: Unit of randomisation
UOA: Unit of analysis
BMI: Body mass index
WHR: Waist hip ratio
F&V: Fruit and vegetables
FFQ: Food frequency questionnaire
QES: Quasi-experimental study
PTPT: Pre-test post-test design
