# Supplementary material for: Effects of worksite health promotion interventions on employee diets: a systematic review
Source: BMC Public Health. 2010 Feb 10;10:62. doi: 10.1186/1471-2458-10-62 (PMC2829502; doi:10.1186/1471-2458-10-62)
Supplement: Additional file 2 — Quality of Included Studies [file 1471-2458-10-62-S2.PDF]

Table 2 - Quality of Included Studies

Randomised, controlled trials (n=10)

	Author, year, country	Control group	Similar baseline characteristics between groups	Randomised		Allocation concealment	Objective outcome measure	Blind assessment	Intention-to-treat analysis	% completing study
				Worksite	Employees					
1	Aldana, 2005<br>USA	√	√		√	√	√	X	√	95
2	Braekman, 1999<br>Belgium	√	√	√		X	√	X	X	82
3	Campbell, 2002<br>USA	√	X	√		X	√	X	√	76
4	De Bourdeaudhuij, 2007<br>Belgium	√	X	√		X	X	X	X<br>(conducted but not reported)	63
5	Emmons, 1999<br>USA	√	√	√		X	X	X	X	42
6	Sorensen 1998<br>USA	√	X	√		X	X	X	X	62
7	Sorensen, 1999<br>USA	√	√	√		X	X	X	√	47
8	Sorensen, 2003<br>USA	√	X	√		X	X	X	X	65
9	Sorensen 2007<br>USA	√	X	√		X	X	X	X	77
10	Steenhuis, 2004<br>Netherlands	√	√	√		X	X<br>(except sales data)	X	X	61

Quasi-experimental studies (n=1)

		Similar baseline characteristics				Objective		Intention-	% completing

	Author, year, country	Control group	between groups	Randomised	Allocation concealment	outcome measure	Blind assessment	to-treat analysis	study
1	Holdsworth, 2004 UK	√	X	X	-	X	X	X	51 (I), 47 (C)

Uncontrolled intervention studies (pre-test post-test design) (n=5)

	Author, year, country	Control group	Similar baseline characteristics between groups	Randomised	Allocation concealment	Objective outcome measure	Blind assessment	Intention-to-treat analysis	% completing study
1	Block, 2004 USA	X	-	X	-	X	-	-	56
2	Calderon, 2008 USA	X	-	X	-	√	-	-	21
3	Holdsworth, 1999 UK	X	-	X	-	X	-	-	100
4	Lassen, 2003 Denmark	X	-	X	-	X	-	-	100
5	Pratt, 2006 17 countries	X	-	X	-	X	-	-	X

√ indicates yes; X indicates no or not reported; - indicates not applicable
I: Intervention
C: Control
