# Supplementary material for: Effects of worksite health promotion interventions on employee diets: a systematic review
Source: BMC Public Health. 2010 Feb 10;10:62. doi: 10.1186/1471-2458-10-62 (PMC2829502; doi:10.1186/1471-2458-10-62)
Supplement: Additional file 3 — Dietary and Anthropometric Outcomes of Included Studies [file 1471-2458-10-62-S3.PDF]

Table 3 - Dietary and Anthropometric Outcomes of Included Studies

Randomised, controlled trials (n=10)

			Change in Anthropometric Measures		Change in Dietary Intakes		
	**Author, year, country**	**Treatment assignment**	**Body weight**	**BMI (kg/m^2)**	**Energy (kcal/day)**	**Total fat**	**Fruit and vegetables (servings/day)**
1	Aldana, 2005<br>USA	I: n = 64<br>C: n = 79	-4.4 kg<br>-1.0 kg	-1.6<br>-0.03	-580<br>-119	-6.7%<br>+1.3%	V: +1.5 F: +0.6<br>V: +0.1 F: 0.0
2	Braekman, 1999<br>Belgium	I: n = 272<br>C: n = 366		Difference between I and C: +0.26	Difference between I and C: -142	Difference between I and C: -1.6%	
3	Campbell, 2002<br>USA	I: n = 282<br>C: n = 256				~ -2.9g/d<br>~ -1.6g/d	V: ~ +0.2 F: ~ +0.5<br>V: ~ -0.1 F: ~ +0.1
4	De Bourdeaudhuij, 2007<br>Belgium	I1: n = 108<br>I2: n = 124<br>C: n = 105				~ -9.1%<br>~ -2.3%<br>~ -1.1%	
5	Emmons, 1999<br>USA	I: n = not reported<br>C: n = not reported				~ -2.2%<br>~ -1.8%	F&V: ~ +0.2<br>F&V: ~ -0.2
6	Sorensen 1998<br>USA	I: 12 sites<br>C: 12 sites				-3.4%<br>-1.6%	F&V: +9%<br>F&V: +4%
7	Sorensen, 1999<br>USA	I1: 7 sites<br>I2: 7 sites<br>C: 8 sites					F&V: +16%<br>F&V: +3%<br>F&V: -2%
8	Sorensen, 2003<br>USA	I: 7 sites<br>C: 8 sites					F&V: -0.1<br>F&V: +0.05
9	Sorensen 2007<br>USA	I: 13 sites<br>C: 13 sites					F&V: +0.37<br>F&V: Not reported<br>(Between-group difference p=0.003)

10	Steenhuis, 2004<br>Netherlands	I1: n = 215<br>I2: n = 290<br>I3: n = 293<br>C: n = 215				~ - 0.4 fat points/d<br>~ -0.3 fat points/d<br>~ +0.1 fat points/d<br>~ 0 fat points/d	V: ~ -13.7g/d F: ~ -0.1<br>V: ~ -7.0g/d F: ~ +0.1<br>V: ~ -10.4g/d F: ~ +0.1<br>V: ~ -1.7g/d F: ~ -0.1<br><br>*Note: F&V intakes at lunch in cafeteria also measured*

Quasi-experimental studies (n=1)

			**Change in Anthropometric Measures**			**Change in Dietary Intakes**	
	**Author, year, country**	**Treatment assignment**	**Body weight**	**BMI (kg/m^2)**	**Energy (kcal/day)**	**Total fat**	**Fruit and vegetables (servings/day)**
1	Holdsworth, 2004<br>UK	I: 4 sites (n = 453)<br><br>C: 2 sites (n = 124)					V: 27% made positive change<br>F: 37% made positive change<br>V: 19% made positive change<br>F: 25% made positive change

Uncontrolled intervention studies (pre-test post-test design) (n=5)

			**Change in Anthropometric Measures**			**Change in Dietary Intakes**	
	**Author, year, country**	**Treatment assignment**	**Body weight**	**BMI (kg/m^2)**	**Energy (kcal/day)**	**Total fat**	**Fruit and vegetables (servings/day)**
1	Block, 2004 USA	I: n = 84				-0.22 times/d consumption of high fat foods	+0.37 times/d consumption
2	Calderon, 2008<br>USA	I: n = 366	~ -3.4 lb	~ - 0.54		-2.3 dietary fat score	
3	Holdsworth, 1999<br>UK	I: n = 12 worksites				Increased sales of healthier foods<br>e.g. polyunsaturated margarine (n=3), low-fat spread (n=5), semi-skimmed milk (n=3), salads (n=3)	
4	Lassen, 2003<br>Denmark	I: n = 5 worksites					F&V: +95g/lunch

5	Pratt, 2006<br>17 countries	I: n = 2498	F&V: Statistically significant increase

I: Intervention
C: Control
BMI: Body mass index
F: Fruit
V: vegetables
